# Sitting on a Sloping Seat Does Not Reduce the Strain Sustained by the Postural Chain

**DOI:** 10.1371/journal.pone.0116353

**Published:** 2015-01-14

**Authors:** Alain Hamaoui, Myriam Hassaïne, Pier-Giorgio Zanone

**Affiliations:** 1 Laboratory of Posture and Movement Physiology, University Champollion, Albi, France; 2 PRISSMH, University of Toulouse, University Paul Sabatier, Toulouse, France; Virginia Commonwealth Univ, UNITED STATES

## Abstract

The objective of this study was to explore the effect of a forward sloping seat on posture and muscular activity of the trunk and lower limbs. To this aim, twelve asymptomatic participants were tested in six conditions varying seat slope (0°, 15° forward) and height (high, medium, low). Angular position of head, trunk and pelvis was assessed with an inertial orientation system, and muscular activity of 11 superficial postural muscles located in the trunk and lower limbs was estimated using normalized EMG. Results showed that a forward sloping seat, compared to a flat seat, induced a greater activity of the soleus (p<0.01), vastus lateralis (p<0.05) and vastus medialis (p<0.05), as well a lower hip flexion (p<0.01). In contrast, no significant variation of head, trunk and pelvis angular position was observed according to seat slope. It was concluded that forward sloping seats increase the load sustained by the lower limbs, without a systematic improvement of body posture.

## Introduction

Following the shift from the 20^th^ century industrial society to nowdays’ digitally-connected society, sitting has become the more common posture at work, and also during the time increasingly devoted to leisure (online games, socializing on the internet…) and learning. Back pain complaints are widespread among people sitting for long periods [1], and awkward seated postures are associated with the risk of developing low back pain [2]. As this musculosketal disorder causes an enormous economic burden on individuals and the economy [3], finding the best posture and seat to reduce the strain (i.e. impairment by overuse) on the spine at its lowest level is an important public health issue.

By Western standards, sitting is considered as an erect posture in which the head and trunk are vertical, the lower legs are bent about 90° at the hips and knees, and the feet are firmly placed on the floor [2]. On the basis of lumbosacral radiographs taken in lateral recumbent position, Keegan [4] revealed that hip flexion was associated with a flattening of the lumbar lordosis, with a very significant rise from 45° to 90°. In the same study, another series of radiographs taken in different seated postures showed that sitting was systematically associated with a flexion of the lumbar spine, whose amplitude increases according to the degree of flexion of the hips. Schoberth [5], who also used an X-ray study, provided evidence that when sitting with the thighs flexed at 90°, only 60° originate from hip flexion, with the other 30° coming from the posterior pelvic tilt. These early works were confirmed by the study of Eklund and Liew [6], who used a flexicurve to assess the lumbar curvature as a function of posture (sitting, lying on the side) and of hip and knee angles. The authors stressed that hip angle is a strong determinant of lumbar posture, with higher hip flexion associated with lower lumbar lordosis.

In their seminal study using *in vivo* recording of intradiscal pressure, Nachemson and Morris [7] showed that L3 and L4 disc pressure was considerably lower in standing posture than in seated posture. In the numerous following studies from the same team (see review in Nachemson et al. [8]), it was also demonstrated that lumbar flexion substantially increases disc pressure in both postures. The effect of lumbar flexion on lumbar disc pressure was later confirmed by Sato et al. [9] and Wilke et al. [10].

Referring to the previously described relation between i) hip flexion and lumbar flexion and ii) lumbar flexion and intradiscal pressure, Mandal [11] [12] [13] [14] has advocated the maintenance of the lumbar lordosis while ssitting, by means of a special seat design. It consisted of a higher chair with a 15° forward tilted seat, which was assumed to open the thigh-trunk angle and to limit the lumbar spine flattening associated with hip flexion.

Although the principle of this “ergonomic” chair was widely disseminated, it was based on few experimental data, coming from photographic observations of clothed people [14]. Bendix and Biering-Sorensen [15], who used statometric measurements, later showed that higher inclination of the seat from 0° to 15° was associated with a more significant lumbar lordosis, but Bendix [16] failed to find significant differences in trunk and head posture between 5° backward and 5° forward tilted seats. Hence, one can assume that the relation between the seat slope and the lumbar posture is not linear neither is it similar across individuals, and that the use of an ergonomic chair does not systematically lead to a more straightened posture.

In addition, forward sloping seats change the direction of the gravity force with respect to the seat top. With flat seats, gravity is perpendicular to the top and only results in pressing the contact area of the body (thighs and buttocks) against the seat. With a forward sloping seat, gravity has an additional component, which is tangential to the seat top and induces a sliding down effect on the pelvis. According to Newton’s first law of motion, this tangential force must be balanced by another force of the same value and acting in the opposite direction, in order to keep the body steady. To our knowledge, no study has so far mapped out the muscles responsible for this action nor their level of activity. However, these parameters could provide qualitative and quantitative information on the strain induced by a sloping seat on the musculoskeletal system. One reason for such a lack of information may be that most studies on sitting posture have focused on the muscular activity of trunk muscles [17] [18] [19] [20] [21] [22] [23], while these muscles are probably located in the lower limbs. Indeed, the sliding down of the pelvis on the sloping seat should result in a flexion of the hips, knees and ankles, which might be counteracted by an overactivity of lower limbs muscles.

The purpose of the present study was to assess the effect of a forward sloping seat on posture and muscular activity of the trunk and lower limbs, using postural angles measurement and surface electromyography.

## Methods

### Participants

Twelve asymptomatic male participants, recruited from the university population, took part in this study. None of them had any recent history of neurological, musculo-skeletal or respiratory disease. The mean (±SD) age, height, weight and body mass index (BMI) were 22 (±3) years, 179(±5) cm, 68.5 (±5) kg, 21.35(±2) kg/m², respectively. Only male subjects, within a reduced range of age, weight and height were selected to build up a homogenous group. Experiments were approved by the local ethics committee (Local Ethics Committee for Human Movement Analysis), and complied with the Helsinki declaration. A written informed consent form was returned by all participants. The individual in this manuscript has given his written informed consent to publish these case details

### Materials

**Electromyography.** A 16-channel wireless EMG device (Zéro Wire model, Aurion, Milan, Italy) was used to quantify the normalized surface electrical activity of the main postural muscles in seated posture with different conditions of seat slope and height.

Eleven superficial muscles of the trunk, neck, shoulder and lower limbs were selected after a pre-test series: neck extensors, trapezius pars descendens, deltoideus pars acromialis, rectus abdominis, erector spinae at T4, T11 and L3 levels, rectus femoris, vastus lateralis, vastus medialis, soleus.

The subjects’ skin was shaved where needed, abraded and cleaned with alcohol to reduce skin impedance to below 5 kΩ. 10 mm diameter (conductive area) Ag/AgCl pre-gelled disposable surface electrodes (PG10S, FIAB, Vicchio, Italy) were applied in a bipolar configuration over the muscle belly in line with muscle fibres direction, on the dominant side of the body. The inter-electrode distance was 20 mm for all sites. All electrode placements were confirmed using palpation and manual resistance tests.

To allow for normalization of the EMGs signals by their maximum values, two 3-s isometric maximal voluntary contractions (MVC) were first carried out for each muscle. Recordings were next taken during the six experimental conditions in sitting posture.

The individual EMG signals were digitized at 1000 Hz using a CompactDAQ with 9215 modules (National Instrument, Austin, USA), controlled by a customed Labview (National Instrument, Austin, USA) program. For each muscle, average rectified EMGs were calculated from the complete recordings, and the values were expressed as a percentage of the data obtained in MVC (normalized EMG).

**Inertial orientation system.** The angular positions of the head, spine, pelvis, and thigh were measured by means of a three-degree-of-freedom orientation inertial system (Inertia Cube3, Intersense Inc., Billerica, USA). It is composed of four wireless trackers (IC3) transmitting data to a USB receiver connected to a computer. Each tracker contains 3 integrated sensing elements in each orthogonal plane: a rate gyroscope, a uniaxial accelerometer and a magnetometer, which measure the angular velocity, the acceleration and the Earth’s magnetic field, respectively. Data from the three sensors are integrated and processed using Kalman filter, to display the orientation as Euler angles (yaw, pitch and roll). The RMS accuracy is 1° in yaw, 0.25° in pitch and roll. Orientation data were collected and synchronized with EMG signals, using a custom-developed Labview program (National Instrument, Austin, USA).

**Figure 1:**
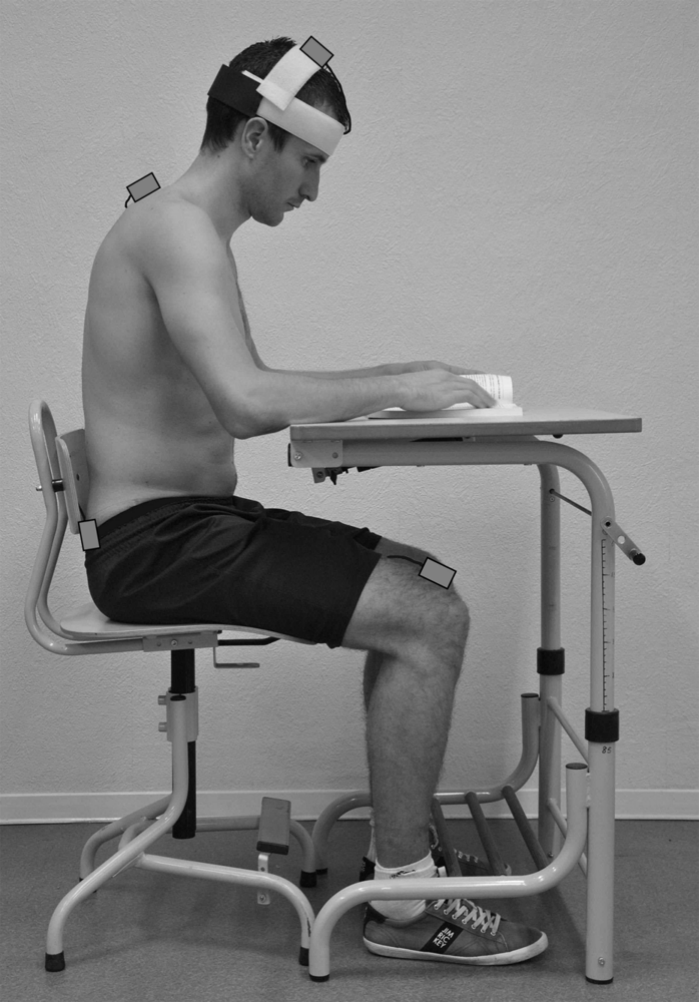
Location of the inertial trackers on the body.

The first tracker was placed on top of the head, at the junction between the two parietal bones, using a system of Velcro bands. The second and the third trackers were adhered to the skin with double sided tape, at the levels of T1 and S1 spinous processes (Fig. 1). The fourth tracker was stuck to the skin on top of the distal extremity of the thigh. These trackers provided data representing the absolute angular position of different body segments in the three orthogonal planes. In this study, which focuses on the effect of a forward sloping seat on posture, only data recorded in the sagittal plane will be presented. Mean values and standard deviations of head flexion (tracker 1), trunk flexion (tracker 2), anterior pelvic tilt (tracker 3) and thigh flexion (tracker 4), were calculated for each trial.

**Figure 2:**
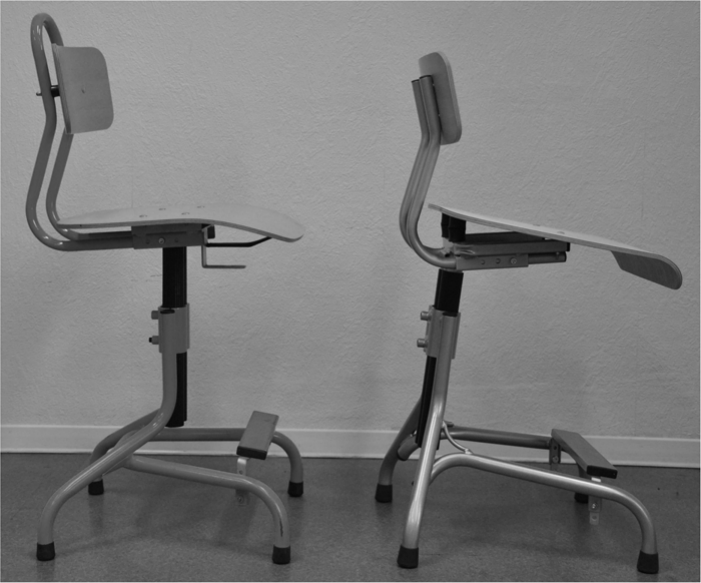
Chair with a flat seat (left) and chair with a 15° forward sloping seat (right).

**Chairs and table.** Two types of chairs (Fig. 2) and one table (Héphaïstos, Rivière sur Tarn, France), which were all height-adjustable were used for the experiment. One chair had a flat seat, and the other, which was specifically designed for the purpose of the experiment, had a 15° forward sloping seat, as recommended by Mandal [11]. The table top was set in a flat position during all tests.

### Procedure

**Anthropometric measurements.** To adjust the seat and table heights according to the anthropometric characteristics of the participants, three parameters were measured prior to testing, using a tape measure:

i) Popliteal height (PH): distance from the floor to the popliteal angle, subject standing barefoot; ii) Elbow height (EH): distance from the seat to the olecranon process, subject seating with elbows flexed at 90°; iii) Shoulder height (ShH): distance from the seat to the acromioclavicular joint.

**Experimental conditions.** For all trials, the participants were asked to adopt the most comfortable posture, while sitting to the back of the chair with their forearms rested on the table. They had to read a short format novel that laid flat on the centre of the table top.

One 5-min recording was taken in 6 different experimental conditions, varying seat slope (no slope, 15° forward slope) and seat height (high, medium, low). A 2-min rest period was given between trials. The order of the experimental conditions was randomly assigned to prevent any order effect.

The three seat height (SH) levels were calculated from the equation of Gouvali and Boudolos [24] (1), which defines the range of recommended seat height.

(PH+2)cos30°≤SH≤(PH+2)cos5° (1)

with SH as the vertical distance from the floor to the highest point on the front of the seat.

The “high” position (Hhigh) was the upper bound of the equation:

Hhigh=(PH+2)cos5°

The “medium” position (Hmed) was the lower bound of the equation:

Hmed=(PH+2)cos30°

The “low” position (Hlow) was the lower bound of the equation minus 10%

Hlow=0.9Hmed

No value was taken beyond the upper bound of the equation, as participants were in this case unable to sit properly to the back of the seat with their feet resting on the floor.

For flat seat (S0 condition), the table height (TH) was set at the upper bound of the equation proposed by Gouvali and Boudolos [24] (2). This value was considered as the most comfortable by the participants in the pre-test series.

THS0=[(PH+2)∗cos5°]+(EH∗0.8517)+(ShH∗0.1483) (2)

with EH as elbow rest height, PH as popliteal height and SH as shoulder height

For forward sloping seat (S15 condition), the desk height was increased by 4.7cm, which is the vertical distance between the front and the rear edge of the seat.

DHS15=DHS0+4.7cm

The data of seat and table heights for each participant are presented in Table 1.

**Table 1:** Seat and table heights (cm) in the different experimental conditions.

	**Hlow(cm)**	**Hmed (cm)**	**Hup (cm)**	**TH S0 (cm)**	**TH S15 (cm)**
**S1**	32	36	41	62.5	67.2
**S2**	39	43	50	75.3	80
**S3**	40	44	51	72.3	77
**S4**	38	42	49	71.4	76.1
**S5**	40	44	51	74.4	79.1
**S6**	38	42	49	63.4	68.1
**S7**	37	41	47	67.4	72.1
**S8**	38	42	49	68.4	73.1
**S9**	36	40	46	63.2	67.9
**S10**	37	42	48	70.4	75.1
**S11**	39	43	50	67.3	72
**S12**	39	43	50	69.4	74.1
**Mean**	**37.8**	**41.8**	**48.4**	**68.8**	**73.5**
**SD**	**2.2**	**2.2**	**2.8**	**4.3**	**4.3**

Seat heights are presented for low (Hlow) medium (Hmed) and high (Hhigh) conditions. Table heights are presented for flat seat (DH S0) and sloping seat (DH S15). Individual values (S1–S12) are displayed, along with means and standard deviations (SD) of the group.

### Data analysis

Two independent variables were considered in this study. i) the seat slope with two levels: flat seat and 15° forward sloping seat. ii) the seat height with three levels: high, medium and low.

Parameters calculated from the EMG device (normalized EMG of 11 muscles), inertial system (mean angular position of four trackers along the sagittal plane) and numeric comfort scale (score from 1 to 5) provided 16 dependent variables.

A two-way repeated-measures analysis of variance (ANOVA) was conducted for each dependent variable, with seat slope and seat height as within-subjects factors. When statistical significance was reached for the seat height factor, the analysis was completed by within-subjects contrasts to compare levels. The significance level was set at p < 0.05. The analysis was performed using Statistical Package for Social Sciences (SPSS) software V14.0 (Chicago, USA).

## Results

### EMG measurements

**Figure 3:**
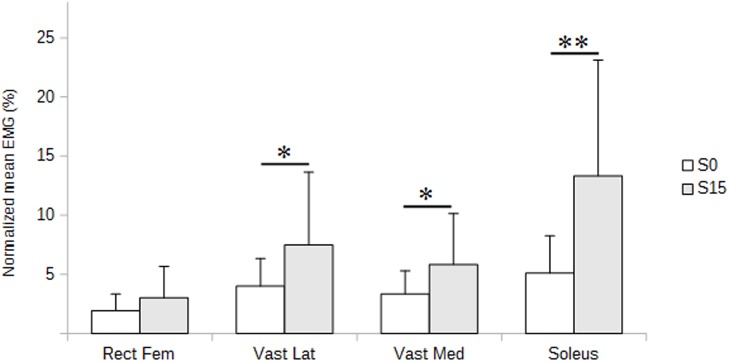
Normalized EMG of lower limbs extensors as a function of seat slope and height. Superfical extensors of knee and ankle are presented: rectus femoris (Rect Fem), vastus lateralis (Vast L), vastus medialis (Vast M), soleus (Sol). Means and standard deviations are illustrated in no sloping (S0) and 15° forward sloping (S15) conditions, with low (Hlow), medium (Hmed) and high (Hhigh) heights settings. *p<0.05, **p<0.01.

**Figure 4:**
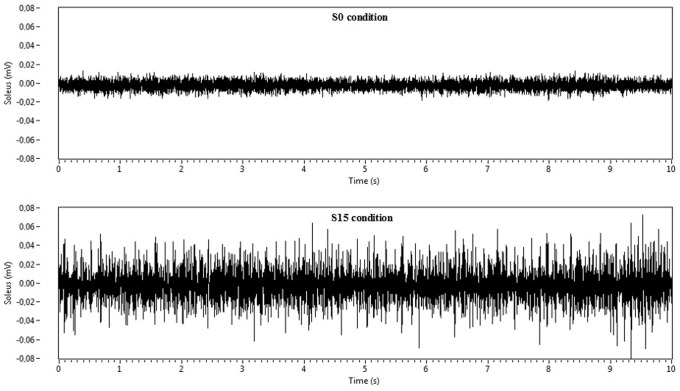
Raw EMGs data of the soleus (mV) when sitting on a flat seat (S0) and on 15° forward sloping seat (S15). Recodings were taken from a representative subject, with the seat height set at the medium level (Hmed).

The most striking result was that normalized EMG of lower limbs extensors was significantly higher when using the 15° forward sloping seat (S15 condition) compared to the flat seat (S0 condition) (Fig. 3). Mean values were 1.5 to 2.5 times greater for vastus medialis (p<0.05), vastus lateralis (p<0.05) and soleus (p<0.01). Although the same trend was observed for rectus femoris, no significant variation could be found. (Table 2). Visual inspection of EMG raw data exhibited higher peak to peak values in S15 condition compared to S0, with a relatively stable difference all along the 5-min trials (Fig. 4).

**Table 2:** Normalized mean rectified EMG as a function of seat slope and height.

	**Neck Ext**	**Trap PD**	**Delt PA**	**ESpi T4**	**ESpi T11**	**ESpi L3**	**Rect Abd**	**Rect Fem**	**Vast L**	**Vast M**	**Sol**
**S0 Hlow**	16.0 ± 13	3.16 ± 2.0	0.86 ± 0.3	4.32 ± 1.8	9.92 ± 5.4	5.27 ± 3.0	2.42 ± 1.5	1.83 ± 1.4	4.00 ± 2.3	3.34 ± 1.9	5.05 ± 3.2
**S0-Hlmed**	16.6 ± 14	2.97 ± 2.5	0.90 ± 0.4	3.69 ± 1.2	10.46 ± 6.0	8.15 ± 4.8	2.44 ± 1.5	1.98 ± 1.5	3.95 ± 2.3	3.35 ± 2.0	5.08 ± 3.2
**S0-Hup**	16.6 ± 15	2.38 ± 2.5	0.92 ± 0.4	4.40 ± 2.2	9.18 ± 6.6	6.95 ± 4.0	2.35 ± 1.4	1.96 ± 1.3	4.04 ± 2.4	3.35 ± 1.9	5.16 ± 3.1
**S15-Hlow**	16.0 ± 13	2.88 ± 2.0	0.90 ± 0.4	3.81 ± 1.3	6.85 ± 5.5	4.02 ± 2.5	2.48 ± 1.5	3.60 ± 3.5	7.20 ± 8.9	5.77 ± 5.9	12.24 ± 9.1
**S15-Hmed**	17.2 ± 15	3.29 ± 3.2	0.94 ± 0.4	3.77 ± 1.4	8.04 ± 5.6	4.71 ± 2.9	2.59 ± 1.8	2.73 ± 1.9	6.71 ± 4.1	5.62 ± 3.9	14.09 ± 10
**S15-Hup**	16.4 ± 14	4.79 ± 6.2	0.89 ± 0.3	4.88 ± 2.1	8.49 ± 5.4	4.14 ± 1.9	2.45 ± 1.5	2.72 ± 2.6	8.56 ± 5.4	6.12 ± 3.1	13.66 ± 9.9
*P(S0/S15)*	**NS**	**NS**	**NS**	**NS**	**NS**	*	*	**NS**	*	*	**
*P(HOverall)*	**NS**	**NS**	**NS**	**NS**	**NS**	*	**NS**	**NS**	**NS**	**NS**	**NS**
*P(Hlow/Hup)*	**NS**	**NS**	**NS**	**NS**	**NS**	**NS**	**NS**	**NS**	**NS**	**NS**	**NS**
*P(Hlow/Hmed)*	**NS**	**NS**	**NS**	**NS**	**NS**	**	**NS**	**NS**	**NS**	**NS**	**NS**

Data from eleven superficial muscles are presented: neck extensors (Neck Ext), trapezius pars descendens (Trap PD), deltoideus pars acromialis (Delt PA), erector spinae at T4 level (ESpi T4), T11 level (ESpi T11) and L3 level (Espi L3), rectus abdominis (Rect Abd), rectus femoris (Rect Fem), vastus lateralis (Vast L), vastus medialis (Vast M), soleus (Sol). The seat height conditions were low (Hlow), medium (Hmed) and high (Hhigh), with no slope (S0) and 15° forward slope (S15). Mean ± SD are expressed as a percentage of the values recorded in the MVC test of each muscle. *p<0.05, **p<0.01, NS (non-significant).

At the trunk level, rectus abdominis displayed greater normalized EMG in S15 than in S0 (p<0.05), but a reversed effect appeared for L3 erector spinae (p<0.05), with lower values in S15 than in S0. Still for L3 erector spinae, the ANOVA revealed an overall effect of seat height (p<0.05), but the subsequent contrast analysis showed only a significant increase in medium condition (Hmed) compared to low condition (Hlow) (p<0.01) (Table 2). No other substantial variation of EMG data was observed as a function of seat height.

### Angular position measurements

**Figure 5:**
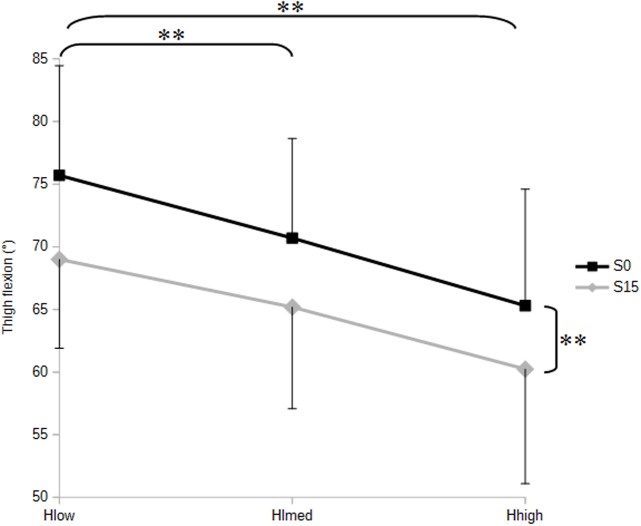
Thigh flexion as a function of seat slope and height. Mean values are illustrated in no sloping (S0) and 15° forward sloping (S15) conditions, with low (Hlow), medium (Hmed) and high (Hhigh) height settings.**p<0.01

Angular position data revealed a decrease of thigh flexion in S15 compared to S0 (p<0.01) (Fig. 5), but no significant variation between S15 and S0 was observed for flexion of the head, trunk and sacrum (Table 3). It should be noted that pooled data across the three seat height conditions exhibited a mean value of S1 flexion, which represents the anterior pelvic tilt, higher in S15 than in S0 condition.

**Table 3:** Angular positions as a function of seat slope and height.

	**Head flexion**	**T1 flexion**	**S1 Flexion**	**Thigh flexion**
**S0 Hlow**	38.1 ± 9	50.0 ± 12	-3.00 ± 7	75.7 ± 9
**S0-Hlmed**	39.9 ± 7	47.3 ± 12	-2.46 ± 4	70.7 ± 8
**S0-Hup**	42.5 ± 11	50.8 ± 14	-1.04 ± 4	65.3 ± 9
**S15-Hlow**	36.3 ± 11	52.0 ± 19	1.47 ± 7	69.0 ± 7
**S15-Hmed**	39.4 ± 9	46.8 ± 12	0.40 ± 6	65.2 ± 8
**S15-Hup**	40.5 ± 10	47.0 ± 12	-2.10 ± 8	60.2 ± 9
*P(S0/S15)*	NS	NS	NS	*
*P(HOverall)*	*	NS	NS	**
*P(Hlow/Hup)*	*	NS	NS	**
*P(Hlow/Hmed)*	NS	NS	NS	**

Mean ± SD of head flexion, T1 flexion, S1 flexion and thigh flexion are presented in different conditions combining seat height (Hlow, Hmed, Hhigh) and slope (S0, S15). Negative values represent an articular position in extension. For the “seat height” independent variable, the overall effect (HOverall) and the contrast analysis between Hlow/Hhigh and Hlow/Hmed are described. * p<0.05, ** p<0.01, NS non-significant.

**Figure 6:**
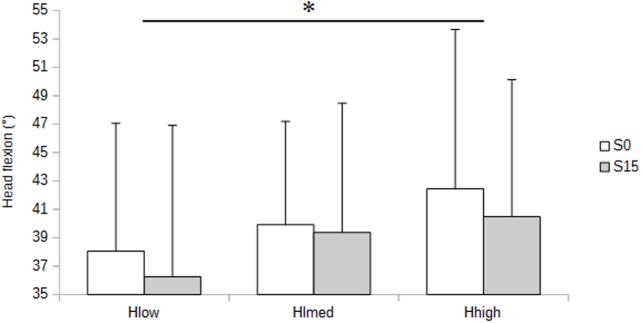
Head flexion as a function of seat slope and height. Mean values are illustrated in no sloping (S0) and 15° forward sloping (S15) conditions, with low (Hlow), medium (Hmed) and high (Hhigh) height settings.

Thigh flexion consistently decreased stepwise from low (Hlow) to medium (Hmed) and high (Hhigh) seats heights (p<0.01) (Fig. 5), while head flexion was greater in Hhigh than in Hlow (p<0.05) (Fig. 6).

No significant variation according to seat height or slope was observed at T1 level.

In addition, participant-by-participant analysis suggested a wide inter-individual variety of trunk and sacrum angular position, which could explain the important standard deviation of the parameters calculated from the whole group. Some participants would for example present the expected flexion of S1 (i.e. an anterior pelvic tilt) in S15, while others exhibited an extension of S1 (i.e. posterior pelvic tilt) in the same condition.

## Discussion

### Forward sloping seat increases the load sustained by the lower limbs

A key finding of the present study is that sitting on a 15° forward sloping seat (S15 condition) compared to flat seat (S0 condition) induces greater muscular activity of the lower limbs extensors, namely, soleus, vastus lateralis and vastus medialis. This phenomon could be related to the tangential component of gravity in S15, which entails a sliding down effect on the body. This effect is likely to be counterbalanced by an increased activity of thigh and lower limbs muscles, which may present 2.5 times greater values compared to S0 condition.

This increase of musuclar activity levels could firstly lead to inner muscular fatigue. For vastus lateralis and vastus medialis, which originate on the patella, it may also increase the load exerted on the femoropatellar joints and on their cartilage. This additional strain on the lower limbs does not support Mandal’s view that the risk of sliding down should be prevented by an ordinary woven seat-cover [11]. Certainly, the choice of a seat cover with an important coefficient of friction could be considered, but it would also restrict the freedom to vary body posture. This may turn out to be harmful in the long run, since it may reduce the variation in pressure distribution on the surface contact area and in the load applied to the different parts of the postural chain. Another way to compensate for the sliding effect is to use a knee/shin support, as in kneeling chairs, but it may also limit the ability to vary posture, with the knees kept flexed at a fixed angle.

### Forward sloping seat induces a different motor pattern at the trunk level

Data from trunk muscles revealed that sitting on a sloping seat induced a higher activity of RA but a reverse effect on ES L3 (p<0.05), suggesting a different muscular pattern between S0 and S15. However, data did do not support the idea of a more straightened posture in S15, which would have required a more consistent activity of postural muscles. Indeed, there is a general consensus in the literature on the fact that upright sitting, compared to relaxed or slumped sitting, is associated with a higher activity of posterior trunk muscles [17] [19] [20] [21] [23] [25] and to a lesser extent of abdominal muscles [23].

No effect of seat slope was observed for muscles located in the upper part of the trunk (neck extensors, trapezius pars descendez, erector spinae at T4 and T11 levels), any more than for the head and T1 angular position. This absence of variation is in line with Bridger’s [26] statement that the postural variables influenced by the chair take place in the lower part of the body.

### Forward seat slope doesn’t systematically straighten trunk posture

In line with EMG examination, the analysis of angular positions did not support the hypothesis of a more straightened posture in the sloping seat condition, as data recorded at the head and trunk levels (head flexion, T1 flexion, S1 flexion) did not display any significant variation between S0 and S15. These findings differ from those of previous studies supporting the use of sloping seats, and describing a more preserved lumbar lordosis and anterior pelvic tilt [14][15] [16] [26]. However, some technical issues question the reliability of these surveys: Mandal [14] used a video analysis with markers fixed on clothes, Bendix [16] found slight differences with no significant variation, and Bridger [26] used a very high seat slope of 25° with a knee pad. These results should therefore be interpreted with caution.

Otherwise, our results showed that pooled data across the three seat height conditions (Hlow, Hmed, Hhigh) exhibited a higher mean value of S1 flexion in S15 condition (not significant), which was also found for Hlow and Hmed but not for Hhigh. Hence, the use of different seat height levels, although calculated from the range of recommended values [24], may have concealed a stronger effect occurring at a specific level.

### Hip flexion is not a strong determinant of pelvic tilt while sitting

Angular position measurements provided evidence that forward sloping seat as well as increased seat height open the thigh-trunk angle, but with no significant effect on anterior pelvic tilt and on trunk posture. These findings are not in line with a study by Keegan [4], who described a close relationship between hip flexion and the flattening of the lumbar lordosis, on the basis of radiographs taken in lateral recumbent. They also contrast with the work of Eklund and Liew [6], who stated that hip flexion is a strong determinant of lumbar posture, as assessed by flexicurve measurements on participants sitting and lying down. Nevertheless, this author also emphasized that gravity tends to decrease extreme lumbar curvature, suggesting that the effect of hip flexion could be lessened when body posture is challenged by the force of gravity. Likewise, one can assume that the effect of hip flexion on trunk posture may also be limited by some comfort criteria, which may be different between lateral recumbent and sitting postures. Because of gravity, keeping an upright posture while sitting requires a higher level of postural muscles activity [17] [19] [21] [27]. This posture could also be unusual for some participants, leading to a feeling of discomfort, as suggested by the lower comfort score in S15 condition. In this case, the effect of hip extension on thigh and gluteal passive muscular tension [4], which drives the pelvis to a more forward tilted position, could be counteracted actively by a different pattern of postural muscles. Since the joints are not fixed in an extreme angular position, the articular free play allows a wide variety of postures on the same seat. As an example, participants were able to keep four different seated postures (slump, flat, long lordosis, short lordosis) while seated on the same stool adjusted at the popliteal height [21]. In the current study, some participants adopted a slumped posture in S15 condition, while others presented a more pronounced lumbar lordosis.

### Maintaining a good posture is not only a matter of seat characteristics

The wide inter individual variety of postures observed in this experiment is consistent with the study by Bridger [26], who described great differences among participants, whether seated on a flat chair or on a forward sloping chair. Although such a variability may be a drawback for those who wish to prevent back pain with furniture design, it lends support to the idea that posture in not only a matter of seat. It is determined by the motor pattern of postural muscles activity, which stabilizes the different bony segments in a given geometrical configuration [28]. Posture is also a question of awarness of one’s own body, which cannot be shaped by the seat settings alone. As a consequence, improving seated body posture should require additional postural education, which will be aimed at developing the sensory-motor abilities needed to adopt and maintain optimal body posture.

Under actual working conditions, the seated posture might also be influenced by other physical (task to perform, ligthing, noise…) and psychological (cognitive load, stress,…) factors, which need to be specifically explored.

### Seat height has an influence on head position

Although the main objective of this study was not to explore the effect of seat height, it must be noted that increased seat height led to greater head flexion (p<0.01). This somewhat predictable finding should be related to the need for keeping a relatively constant distance between the eyes and the book to limit eye accommodation. Head and neck flexion should thus compensate for the lengthening of the eye-to-object distance due to a higher position of the seat.

In contrast with head flexion, EMGs of upper trapezius and neck extensors did not vary with seat height, suggesting a limited effect on muscular activity or variations occurring in deeper muscles.

### Limitations

There are some limitations to the study which need to be acknowledged. Firstly, this survey only focused on seat height and slope, and did not explore any interaction with other characteristics of the work station, such as seat depth, backrest shape and height. Secondly, all participants were young male subjects with a normal body mass index, and some variations may occur with other types of population. Finally, all recordings were taken during a reading task in a laboratory environment, while other usual situations such as writing, drawing or typing on a computer may have yielded to some different results. These factors could conceivably be tested in future studies, provided a time limit is set beforehand, to prevent fatigue and lassitude.

## Conclusion

The use of a forward sloping seat cannot be considered as a reliable means of improving body posture, in particular to preserve anterior pelvic tilt and lumbar lordosis while seated. Conversely, it requires a greater muscular activity of knee and ankle extensors, leading to an increased load on the lower limbs.
